# Parthenolide inhibits the progression of intrahepatic cholangiocarcinoma by promoting ferroptosis through inhibiting UBD

**DOI:** 10.1080/15384047.2026.2664327

**Published:** 2026-04-28

**Authors:** Yuanzhu Xie, Jia Wang, Jia Zhou, Yuhuai Peng, Yizhi Wang, Yueren Wang, Yinghui Song

**Affiliations:** a Central Laboratory, Hunan Provincial People's Hospital/The First Affiliated Hospital Hunan Normal University, Changsha, China; b Institute of Emergency Medicine, Hunan Provincial People's Hospital/The First Affiliated HospitalHunan Normal University, Changsha, China; c Scientific Research Office, Hunan Provincial People's Hospital/The First Affiliated HospitalHunan Normal University, Changsha, China; d Department of Hepatobiliary Surgery, Hunan Provincial People's Hospital/The First Affiliated HospitalHunan Normal University, Changsha, China

**Keywords:** Parthenolide, intrahepatic cholangiocarcinoma, UBD, ferroptosis, HMOX-1

## Abstract

**Objective:**

This study aimed to investigate the antitumor activity and molecular mechanism of Parthenolide (Par) against intrahepatic cholangiocarcinoma (ICC), a highly malignant liver tumor with poor prognosis, to explore potential new treatment strategies.

**Methods:**

Par was identified through screening an FDA compound library. *In vitro* assays were performed to assess its effects on ICC cell proliferation, colony formation, invasion, migration, cell cycle, and apoptosis. An *in* *vivo* nude mouse tumor model was used to evaluate tumor growth inhibition. Transcriptomic analysis, molecular docking, and cellular thermal shift assays were employed to identify key targets and pathways. Changes in ferroptosis-related markers, including iron ion levels, lipid peroxides, SOD activity, and GPX4 expression, were measured. Rescue experiments with the ferroptosis inhibitor ferrostatin-1 were conducted to validate the role of ferroptosis.

**Results:**

Par significantly inhibited ICC cell proliferation, colony formation, invasion, and migration, induced G2/M phase arrest, and promoted apoptosis *in vitro*; and suppressed tumor growth *in vivo*. Transcriptomic analysis showed upregulation of HMOX-1 and downregulation of Ubiquitin D (UBD) after Par treatment. Par was confirmed to bind to the UBD protein. Enrichment analysis indicated ferroptosis as a key pathway, with Par treatment leading to increased iron ions, lipid peroxide accumulation, reduced SOD activity, and downregulated GPX4. Ferrostatin-1 partially reversed Par-induced inhibitory effects.

**Conclusion:**

This study demonstrates for the first time that Par exerts anti-ICC effects related to inhibition of UBD, thereby activating the ferroptosis pathway. These findings provide a novel potential strategy and therapeutic target for ICC treatment.

## Backgrounds

Intrahepatic cholangiocarcinoma (ICC) is the second largest primary liver malignancy after hepatocellular carcinoma, originating from the epithelial cells of the intrahepatic bile duct.^1^ In recent years, the global incidence rate and mortality of ICC have continued to increase, posing a serious public health challenge.^2^ Due to its concealed anatomical location and atypical early symptoms, most patients with ICC are diagnosed late and miss the opportunity for radical surgery.^3^ Although systemic chemotherapy with gemcitabine combined with cisplatin is the first-line standard regimen for advanced ICC, its objective response rate is limited, and drug resistance is almost inevitable. The median survival of patients rarely exceeds 1 y.^4^^,^^5^ Targeted therapies, such as targeting IDH1 mutations, FGFR2 fusion, etc., and immune checkpoint inhibitors, have brought hope to some patients, but their applicability is limited by specific genotypes, resulting in limited overall benefits.^6^ Therefore, exploring the new mechanisms underlying the occurrence and development of ICC, and developing new, efficient, and broad-spectrum treatment strategies based on this, has become a core challenge that urgently needs to be overcome in current clinical and basic research.

With the continuous deepening of cell death research, ferroptosis, a novel regulatory cell death mode driven by excessive accumulation of lipid peroxides and dependent on iron, has been shown to be a key node in tumor suppression.^7^^,^^8^ Unlike apoptosis, ferroptosis has unique morphological and biochemical characteristics, and it remains effective against many tumor cell lines that have developed resistance to traditional apoptosis inducers, making it an attractive new target for tumor therapy.^9^ Inducing ferroptosis can inhibit the invasion and metastasis ability of ICC cells.^10^ ICC cells can inhibit ferroptosis through the PAX8-AS1/GPX4 signaling axis, leading to resistance to gemcitabine and cisplatin.^11^ The use of GPX4 inhibitors combined with chemotherapy can significantly enhance the anti-tumor effect in preclinical models.^12^ The combination of sorafenib and photodynamic therapy was also found to induce ferroptosis by regulating ACSL4 and GPX4, thus inhibiting cancer cells.^13^ Therefore, targeted ferroptosis is not only expected to reverse chemotherapy resistance in ICC but also can be used to develop new combination therapy regimens, making it a highly promising research direction in this field.

Natural products have always been a treasure trove for the development of anti-tumor drugs because of their structural diversity and rich biological activity. Parthenolide is a sesquiterpene lactone compound isolated from the traditional medicinal plant Tanacetum parthenium, which has been used for the treatment of migraine and inflammatory diseases for a long time.^14^ In the past two decades, research has found that Parthenolide has a broad-spectrum and a strong antitumor potential, and its mechanism of action involves inducing cell cycle arrest, promoting apoptosis, inhibiting key survival signaling pathways such as the NF-κB and STAT3, and regulating tumor stem cell characteristics.^15^^,^^16^ However, previous studies focused mainly on leukemia, breast cancer, glioma, and other tumors.^17^ The mechanism of the effect of Parthenolide in the treatment of cholangiocarcinoma has also attracted attention. There is a study that illustrates the effects and mechanisms of Parthenolide in human extrahepatic cholangiocarcinoma cells TFK1 through a metabolomics approach based on liquid chromatography/mass spectrometry (LC/MS).^18^ The effect of parthenolide on cholangiocarcinoma has also been reported to depend on apoptosis by Bax translocation.^19^ Parthenolide is also believed to promote apoptosis of cholangiocarcinoma cells by inhibiting COX2 and PKC-alpha.^20^^,^^21^ However, currently there is no report on whether Parthenolide can induce ferroptosis in ICC cells and the possible mechanism. Based on this, this study explores the effect and potential mechanism of Parthenolide on ICC through experimental systems *in vivo* and *in vitro*, providing a new theoretical basis and potential therapy for ICC.

## Methods and materials

### Cell line

Human ICC cell lines, including HuCCT1 (#SCSP-5480), HCCC9810 (#SCSP-556), and RBE (#SCSP-557) in this study, were purchased from the NCACC cell bank (National Collection of Authenticated Cell Cultures). Cells were cultured in RPMI-1640 medium (HyClone) containing 10% fetal bovine serum (FBS, Gibco) and 1% penicillin/streptomycin (Gibco) in a constant temperature and humidity incubator at 37 °C and 5% CO_2_. All cell lines were validated through STR analysis (NCACC cell bank), and routine mycoplasma detection was performed using the universal mycoplasma detection kit (#C0301S, Beyotime).

### Regents

The FDA-approved drug library (#L1300), Parthenolide (Par, S2341), and the feroptosis inhibitor (Ferrostatin-1, Fer-1, S7243) were purchased from Selleck Chemicals LLC (Houston, Texas, USA). The CCK-8 reagent kit (#CK04) was purchased from Dojindo (Kumamoto Prefecture, Japan). The Annexin V/FITC apoptosis detection kit (#LHK601-050-*P*) was purchased from Jiamay Biotech Co., Ltd. (Beijing, China). MDA detection kit (#S0131S) and SOD activity detection kit (#S0101S) were purchased from Beyotime Biotech Co., Ltd. (Hangzhou, China). The iron assay kit (#ab83366) was purchased from Abcam (Cambridge, UK).

### Animals

Male BALB/c nude mice aged 4–6 weeks and weighing 18–20 g were purchased from Changsha Slack Jingda Experimental Animal Technology Co., Ltd. and housed in SPF-grade animal rooms, with a 12-h light–dark cycle and free access to food and water. All animal experiments follow the 3 R principle (reduce, optimize, replace), and the use and care of the mice for this study were reviewed and approved by the Institutional Animal Committee of the Hunan Provincial People's Hospital (SYXK-Xiang-2024-0001).

### CCK-8 assay

Cell viability of ICC cells treated with Par was assessed using a CCK-8 assay. Cells in the logarithmic growth phase were seeded in 96-well plates at a density of 5 × 10³ cells per well. After cells were cultured overnight, the medium was then replaced with fresh medium containing varying concentrations of Par (0, 1.25, 2.5, 5, 10, 20 µM). Following a 24-h incubation, 10 µL of CCK-8 solution was added to each well, and the plates were further incubated for 2 h. Absorbance at 450 nm was measured using a microplate reader.

### Clone formation experiment

Clonogenic survival was assessed using a colony formation assay. Briefly, cells in the logarithmic growth phase were seeded at a low density of 500 cells per well in 6-well plates. After overnight attachment, the medium was replaced with fresh medium containing Par at specified concentrations (0, 5, 10 µM). The drug-containing medium was renewed every 3 d. After 10–14 d of incubation, when visible colonies appeared in the control wells, the experiment was terminated. The colonies were fixed with 4% paraformaldehyde for 15 min, stained with 0.1% crystal violet for 30 min, and then photographed for documentation. A cluster of 50 or more cells was defined as a colony and counted for subsequent statistical analysis.

### Cell cycle analysis

The effect of Par on the cell cycle was analyzed by flow cytometry. Following a 24-h treatment with various concentrations of Par, cells were harvested by trypsinization, washed, and fixed overnight at 4 °C with pre-cooled 70% ethanol. Fixed cells were then washed with PBS and stained in the dark for 30 min using a solution containing 100 µg/mL RNase A and 50 µg/mL propidium iodide (PI). Cell cycle distribution (G0/G1, S, and G2/M phases) was determined with a Beckman flow cytometer, and the data were analyzed using dedicated software.

### Cell apoptosis analysis

Apoptosis was detected using flow cytometry with Annexin V-FITC/PI dual staining. Following treatment, cells were harvested by trypsinization (without EDTA), washed twice with ice-cold PBS, and resuspended in 1 × Binding Buffer according to the manufacturer's protocol. The cell suspension was then stained with Annexin V-FITC and Propidium Iodide (PI) and incubated in the dark at room temperature for 15 min. The samples were analyzed by flow cytometry in 1 h. Cells were categorized as follows: viable (Annexin V−/PI−), early apoptotic (Annexin V+/PI−), and late apoptotic/necrotic (Annexin V+/PI+). The total apoptosis rate was calculated as the sum of the percentages of early and late apoptotic cells.

### Transwell experiment

Cell migration was assessed in a transwell assay. Briefly, a cell suspension containing 5 × 10⁴ cells and the specified concentration of Par in serum-free medium was added to the upper chamber (200 µL). The lower chamber was filled with 600 µL of complete medium supplemented with 10% FBS to serve as a chemoattractant. After incubation, nonmigratory cells on the upper surface of the membrane were removed with a cotton swab. Migrated cells on the lower surface were fixed with 4% paraformaldehyde, stained with crystal violet, and manually counted in five randomly selected microscopic fields per well.

### Wound healing assay

Cell migration was assessed using a standard scratch assay. HuCCT1 cells were seeded in six-well plates at a density of 5 × 10⁴ cells per well. Upon reaching the appropriate confluency, a straight scratch was generated across the monolayer using a sterile pipette tip. The detached cells were removed by gently washing them with PBS. The medium was then replaced with serum-free or low-serum culture medium to minimize cell proliferation. The plates were incubated for 24 h, after which the scratch areas were observed and photographed under a microscope. The degree of cell migration into the wound area was determined by measuring and comparing the changes in the scratch width.

### Nude mouse subcutaneous transplantation tumor model

Collect HuCCT1 cells in logarithmic growth phase and resuspend them in serum-free medium. Each nude mouse was subcutaneously injected with 100 μL of cell suspension (containing 5 × 10^6^ cells) in the right back. When tumor volume reached about 50 mm³, tumor bearing mice were randomly divided into three groups (*n* = 5/group): the control group (intraperitoneal injection of physiological saline containing 0.1% DMSO), the Par treatment group (intraperitoneal injection of Par, 5 mg/kg), the Cisplatin (cDDP) group (intraperitoneal injection of Cisplatin (cDDP), 5 mg/kg). The treatment was administered twice a week for 4 weeks. The tumor volume was calculated by the formula volume = (L × W²)/2. The mice were euthanized by cervical dislocation, and the tumor tissue was completely removed and weighed. At the same time, the liver and kidneys were taken and fixed with 4% paraformaldehyde for paraffin embedding and section staining.

### Transcriptome sequencing (RNA seq) and analysis

HuCCT1 cells were treated with 5 μM Par or an equal volume of DMSO as a control for 24 h, with three biological replicates per condition. Total RNA was extracted using TRIzol reagent (Invitrogen). Quality‐checked RNA samples were sent to Beijing Novogene Technology Co., Ltd., for library preparation with the NEBNext® UltraTM II RNA Library Prep Kit and sequencing on the Illumina NovaSeq 6000 platform (paired‑end 150 bp). The raw reads were aligned to the human reference genome (GRCh38) with HISAT2, and the gene counts—were obtained using featureCounts. Differential expression analysis was carried out with the DESeq2 R package, applying thresholds of |log₂(FoldChange)| > 1 and adjusted *p*‑value (*p*adj) < 0.05 to identify significantly changed genes. Gene Ontology (GO) enrichment and Kyoto Encyclopedia of Genes and Genomes (KEGG) pathway analyses were subsequently performed on the differentially expressed genes.

### Real-time fluorescence quantitative PCR (qPCR)

Total RNA was reverse transcribed into cDNA using the PrimeScript RT reagent kit (Takara). Using cDNA as a template, amplification was performed on the QuantStudio 6 Flex system using SYBR Premix Ex Taq II (Takara). The reaction procedure was: pre-denature at 95 °C for 30 s; 95 °C for 5 s, 60 °C for 30 s, cycle 40 times. Using GAPDH as an internal reference gene, the relative expression level of the target gene was calculated using the 2^−ΔΔCt^ method. The primer sequence was synthesized by Biotechnology Co., Ltd. (Supplementary Table 1).

### Western blot

The total protein from the cells was extracted using RIPA lysis buffer and the concentration was determined using the BCA method. Equivalent protein samples were separated by 10%–12% SDS-PAGE electrophoresis and transferred to a PVDF membrane. After being sealed at room temperature for 1 h with 5% skim milk, it was incubated overnight at 4 °C with the primary antibody (dilution ratio shown in Supplementary Table 2). After washing with the TBST, the membranes were incubated with the corresponding HRP-labeled secondary antibody at room temperature for 1 h. Finally, the ECL chemiluminescence assay kit was used to develop on a chemiluminescence imaging instrument. Homogenization treatment was performed using GAPDH as an internal reference.

### Molecular docking

The three-dimensional structure of the human UBD protein (AlphaFold entry O15205) was retrieved from the RCSB Protein Data Bank. The preparation of the receptor and ligand—including hydrogen addition and charge assignment—was conducted with AutoDock Tools 1.5.6, using the 3D structure of the PTL obtained from PubChem. A semiflexible docking approach based on the Lamarckian genetic algorithm (LGA) was employed, with the grid center positioned around the predicted binding pocket. The conformation exhibiting the lowest calculated binding free energy was identified as the most favorable binding mode and subsequently visualized in PyMOL.

### Cell thermal displacement experiment (CETSA)

HuCCT1 cells were treated with 5 μM Par or an equal volume of DMSO for 4 h. Cells were collected, resuspended in PBS, and divided equally into PCR tubes. A PCR instrument was used to perform gradient heating on the sample (46 to 56 °C, with an interval of 2 °C). Immediately after heating, all samples were placed on ice to cool, and then the cells were lysed by repeated freezing and thawing three times. The supernatants (containing proteins with altered thermal stability) were centrifuged, and a Western blot was performed to detect the residual amount of UBD protein at different temperatures.

### Detection of malondialdehyde (MDA)

An MDA detection kit using the thiobarbituric acid method was used to determine the content of MDA according to the instructions. The cells were lysed, and the supernatant was collected. The MDA content was expressed in μmol/mg protein.

### Detection of superoxide dismutase (SOD) activity

The total SOD activity detection kit was used using the WST-8 method to determine SOD activity according to the instructions strictly according to the instructions. The cells were lysed and the supernatant were collected. The total SOD activity was expressed in U/mg of protein.

### Iron ion detection

The intracellular ferrous ion content (Fe2+) was measured using an iron assay kit according to the manufacturer's protocol. Briefly, harvested cells were washed with ice-cold PBS and homogenized on ice with five volumes of iron assay buffer. The homogenate was centrifuged at 13,000 × g for 10 min at 4 °C to pellet insoluble debris. The resulting supernatant was collected, treated with an iron reducer, thoroughly mixed, and incubated at room temperature for 30 min. Subsequently, 100 μL of iron probe was added to each sample. After mixing, the reaction mixture was incubated for 60 min at room temperature in the dark. Absorbance was finally recorded at 593 nm using a microplate reader.

### Cell electron microscopy

The cells were fixed with glutaraldehyde and osmium tetroxide to preserve ultrastructure, then dehydrated with ethanol or acetone, and embedded in epoxy resin; after ultrathin sectioning, the sections were stained with uranyl acetate and lead citrate, and finally observed and imaged under an electron microscope.

### Molecular dynamics simulation

Molecular dynamics simulations were performed using GROMACS 2022.3. Prior to simulation, small molecules were prepared with AmberTools22 for the assignment of the GAFF force field, while hydrogenation and RESP electrostatic potential calculations were conducted using Gaussian 16 W. The derived potential data were subsequently integrated into the molecular dynamics topology files. The simulations were conducted under constant temperature (300 K) and pressure (1 bar). The Amber99SB-ildn force field was applied, with solvation handled using the TIP3P water model. The system charge neutrality was maintained by adding sodium ions (Na⁺). System equilibration included energy minimization via the steepest descent algorithm, followed by 100,000 steps each of NVT and NPT ensemble equilibration with a coupling constant of 0.1 ps, totaling 100 ps per phase. The production dynamics were then run for 100 ns (5 million steps, 2 fs per step). Trajectory analysis was performed using built-in GROMACS utilities to calculate root-mean-square deviation (RMSD), root-mean-square fluctuation (RMSF), radius of gyration and was combined with MM/GBSA free energy estimations and free energy landscape visualizations.

## Cell transfection

The cell transfection was performed by LipofectamineTM 3000 reagent (Invitrogen, #L3000015) according to the instructions. The siRNA and plasmid were purchased from Miaoling Bio (Wuhan, Hubei, China). The sequences for siRNA and plasmid were shown in Supplementary Table 3. The transfection efficiency was confirmed by qRT‒PCR and Western blot.

### Statistical analysis

All *in vitro* experiments were independently repeated at least three times. Data were expressed as mean ± standard deviation (Mean ± SD). A two-tailed Student's t-test was used for comparisons between two groups, while one-way ANOVA was applied for comparisons among multiple groups. Following ANOVA, Tukey's HSD (honestly significant difference) test was employed for post hoc multiple comparisons. All the statistical analyzes were performed with the GraphPad Prism 9.0 software, and *p* < 0.05 was considered statistically significant (*p < 0.05, **p < 0.01, ***p < 0.005, ****p < 0.001).

## Result

### Parthenolide has a significant inhibitory effect on ICC cells based on the screening of the FDA-approved drug library

**Figure 1.**
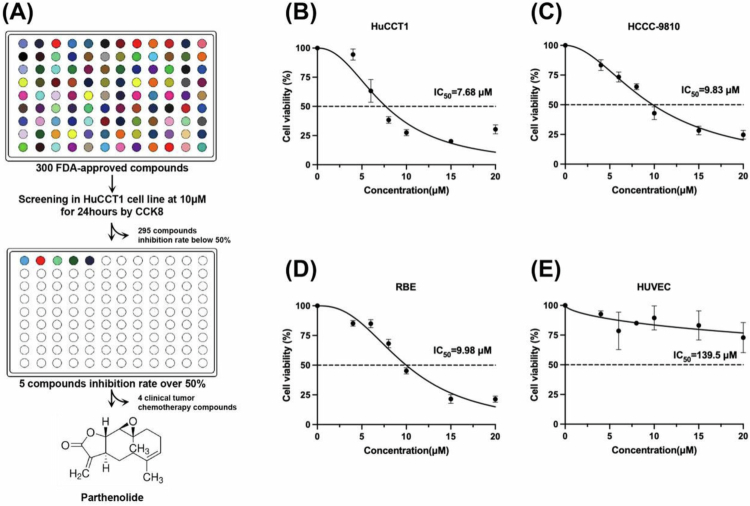
Parthenolide has a significant inhibitory effect on ICC cells based on the screening of the FDA-approved drug library. (A) A high-throughput screening of an FDA-approved drug library identified Par as a candidate compound that significantly inhibited the proliferation of HuCCT1 cells. (B‒D) Dose‒response curves demonstrate that Par inhibits the viability of multiple HuCCT1, HCCC-9810, and RBE in a concentration-dependent manner after 24-h treatment, measured by the dose–response assay. (E) Dose‒response curves demonstrate that Par does not have an obvious inhibition effect on the HUVEC cell. Note: Par: Parthenolide.

To find potential drugs for ICC treatment, we conducted a systematic screening of the FDA-approved drug library. The CCK8 method was used to detect the inhibitory effect of the 10 μM drug on the proliferation of HuCCT1 cells. When screening for 300 drugs, it was found that only 5 drugs could exert an inhibitory effect greater than 50%, of which 4 were already reported clinical chemotherapy drugs. More importantly, we found that the traditional herbal extract, Par, exhibited significant inhibitory effects on HuCCT1 (Figure 1A). Subsequently, we conducted further experiments using more ICC cell lines and used the CCK8 method to detect the growth inhibitory effect of Parthenolide on cells after 24 h of treatment. The results showed that Par inhibited ICC cell viability in a dose-dependent manner (Figure 1B-D). After 24 h of treatment with Par, the half maximal inhibitory concentrations (IC50) on HuCCT1, HCCC-9810, and RBE cells were (7.68 ± 0.42) μM, (9.83 ± 0.31) μM, and (9.98 ± 0.37) μM, respectively. At the same time, we used HUVEC as normal control cells and found that after 24 h of treatment with Par, its IC50 value for HUVEC cells was (139.5 ± 7.80) μM (Figure 1E). These results indicate that Par is an effective inhibitor of ICC cell proliferation.

### Parthenolide inhibitors of ICC cells

**Figure 2.**
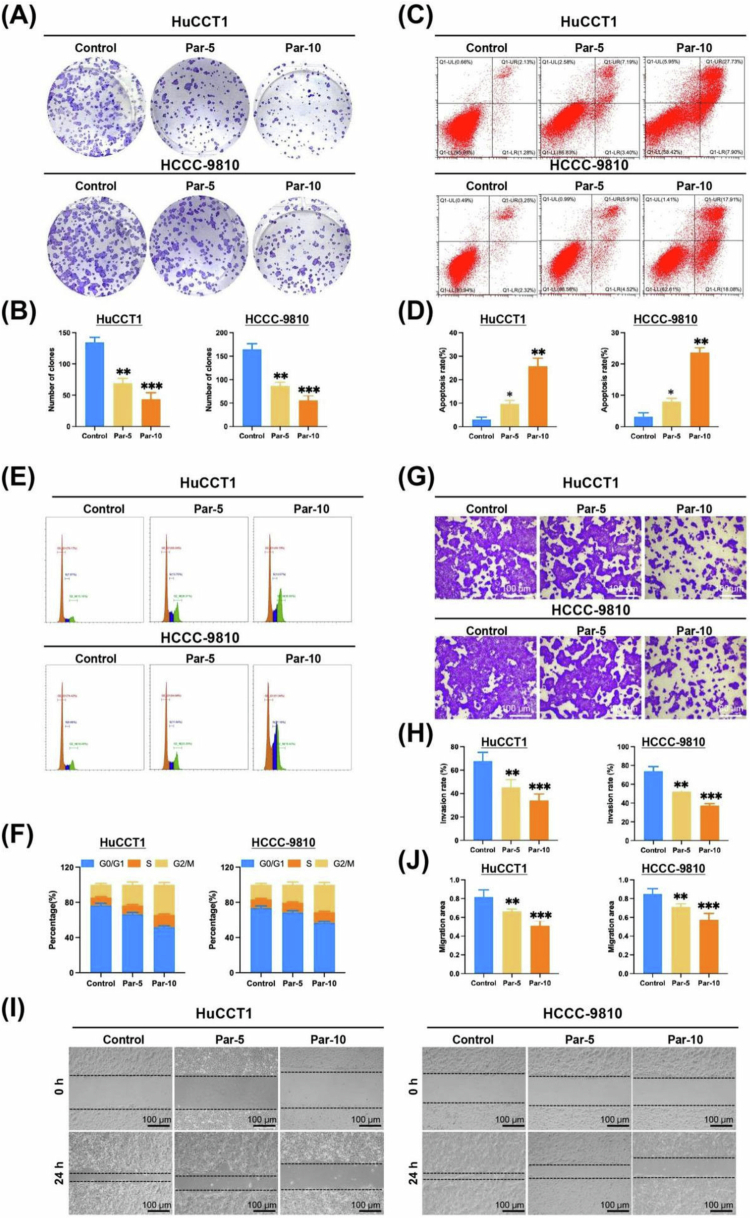
Parthenolide inhibits ICC cells. (A, B) Colony formation assays demonstrate that treatment with Par (5 and 10 µM) significantly reduces the clonogenic survival of HuCCT1 and HCCC-9810 cells. (C, D) Apoptosis analysis by Annexin V-FITC/PI staining shows that Par treatment significantly increases the rate of apoptosis in ICC cells in a dose-dependent manner. (E, F) Cell cycle analysis by flow cytometry reveals that Par treatment induces a phase arrest of G2/M in ICC cells, accompanied by a corresponding decrease in the population of phase G0/G1. (G, H) Transwell invasion assays indicate that Par treatment markedly inhibits the invasive capability of ICC cells. (I, J) Wound healing (scratch) assays show that Par treatment significantly impairs the migration ability of ICC cells. Note: Par: Parthenolide; compared to the Control group, *p < 0.05, **p < 0.01, ***p < 0.005.

To further evaluate the antitumor effect of Parthenolide, we treated HuCCT1 cells and HCCC-9810 cells with Par at 5 and 10 μM. The cloning formation assay showed that Par treatment significantly reduced the number of ICC cell clones (Figure 2A and B). Annexin V-FITC/PI staining method was used to detect cell apoptosis, and Parthenolide treatment significantly increased the apoptosis rate of ICC cells (Figure 2C and D). Through flow cytometry analysis of the cell cycle, we found that Par treatment significantly increased the proportion of cells in the G2/M phase, while the proportion of cells in the G0/G1 phase decreased accordingly (Figure 2E and F). The results of the Transwell experiment showed that Par treatment inhibited the invasion ability (Figure 2G and H). The results of the wound healing assay showed that Par treatment inhibited the migration ability of ICC cells (Figure 2I and J). All of the above results collectively indicate that Par exerts its anti-ICC effect by inhibiting cell proliferation and invasion, blocking the cell cycle, and inducing apoptosis.

### Parthenolide effectively inhibits ICC *in vivo*.

**Figure 3.**
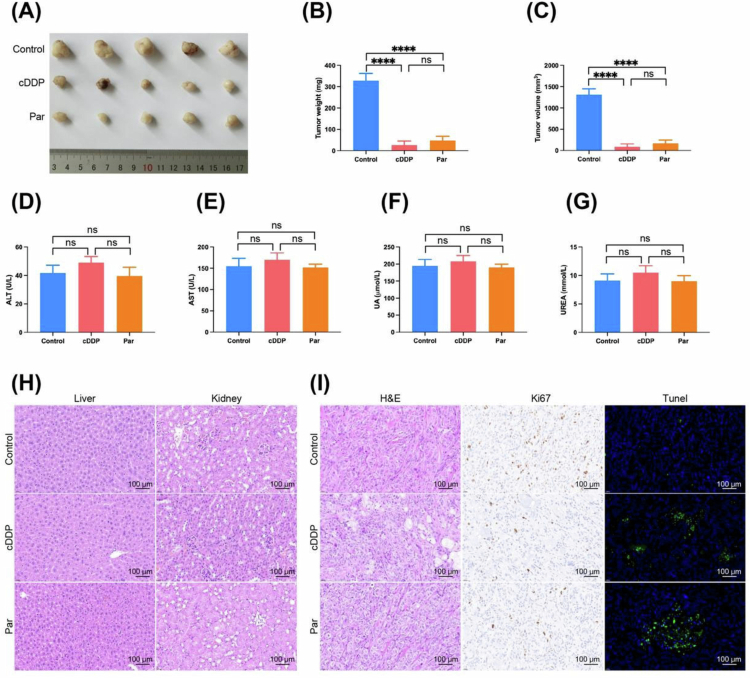
Parthenolide effectively inhibits ICC *in vivo*. (A–C) Par treatment significantly reduces tumor size (representative images in A), tumor weight (B), tumor volume (C) of HuCCT1 subcutaneous xenografts in nude mice compared to the vehicle control group, achieving comparable efficacy to cisplatin (cDDP). (D–G) The biochemical analysis of blood shows no significant changes in key markers of liver (AST, ALT) and kidney (BUN, CRE) function after Par treatment. (H) Hematoxylin and eosin (H&E) staining of liver and kidney tissue sections reveals no significant pathological damage, further supporting the safety of Par treatment. (I) Immunohistochemical staining for Ki67 (proliferation marker) and the TUNEL assay (apoptosis marker) of tumor sections demonstrates that Par treatment significantly inhibits tumor cell proliferation and promotes apoptosis *in vivo*. Note: Par: Parthenolide; scale bar = 100 μm; Compared to Control group, ****p < 0.001.

To validate the *in vivo* efficacy of Par, we constructed a nude mouse subcutaneous transplant tumor model of HuCCT1 cells. After 28 d of treatment, compared to the control group, tumor size, weight, and volume in the Par treatment group were significantly reduced (*p* < 0.001) (Figure 3A‒C), which was comparable to the efficacy of the cDDP treatment group (*p* > 0.05). By detecting liver function and kidney function levels in the blood, it was found that there was no significant change in liver and kidney function in mice at this dose and course of treatment (*p* > 0.05) (Figure 3D‒G), And H&E staining of the liver and kidneys showed no significant pathological damage (Figure 3H), indicating that Par has good *in vivo* safety at this dose. Ki67 staining and tumor TUNEL staining showed that Par can inhibit tumor cell proliferation activity and increase the occurrence of cell apoptosis (Figure 3I).

### The transcriptome reveals that parthenolide inhibits UBD and activates HO-1

**Figure 4.**
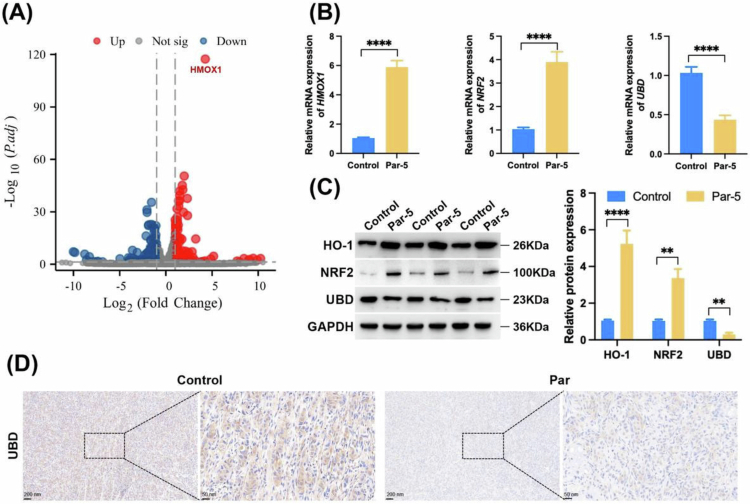
Transcriptome reveals that parthenolide inhibits UBD and activates HO-1. (A) Volcano plot of RNA-sequencing data from HuCCT1 cells treated with Par versus control. Red dots represent significantly upregulated genes (*n* = 150), blue dots represent significantly downregulated genes (*n* = 218). (B, C) Validation of RNA-seq findings by qRT‒PCR (B) and western blot (C) in HuCCT1 cells. Par treatment downregulates UBD expression while upregulating NRF2 and its downstream target HO-1 at both mRNA and protein levels. (D) Immunohistochemical staining confirms that *in vivo* Par treatment significantly downregulates UBD protein expression in HuCCT1 subcutaneous xenograft tumors. Note: Par: Parthenolide; compared to the Control group, **p < 0.01, ****p < 0.001.

To further explore the mechanism of action of Par, we conducted RNA seq analysis on HuCCT1 cells treated and control with Par. Among them, 150 genes were significantly upregulated and 218 genes were significantly downregulated. Figure 4A shows the volcano plot of differentially expressed genes. All differentially expressed genes were included in Supplementary Table 3. Among them, heme oxygenase-1 (HO-1, encoded by the HMOX1 gene) was one of the genes with the most significant upregulation, while UBD was the gene with the most significant downregulation. Due to numerous studies that indicate that Nrf2 can activate upregulation of HO-1, while UBD can release NRF2 inhibition through ubiquitination.^22^^,^^23^ We speculated that Par may activate HO-1 by inhibiting UBD. For this purpose, we used qRT–PCR and Western Blot to detect the levels of mRNA and proteins of UBD, NRF2, and HO-1. We found that Par treatment decreased UBD and upregulated Nrf2 and HO-1 levels (Figure 4B and C). Furthermore, we used immunohistochemistry to detect UBD levels in tumors of nude mice, which were consistent with the trend of changes at the cellular level (Figure 4D).

**Figure 5.**
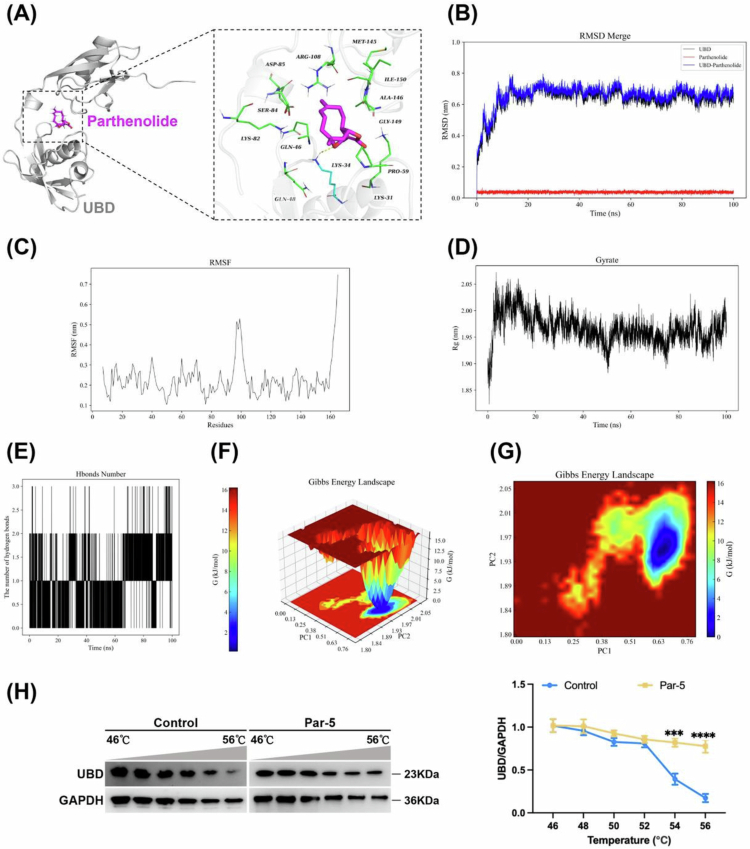
Parthenolide could inhibit UBD expression. (A) Molecular coupling shows that Par occupies a hydrophobic pocket on UBD with a binding energy of −6.768 kcal/mol. (B–D) 100  ns molecular dynamics simulations confirm the stability of the UBD-Par complex: Root-mean-square deviation (RMSD) indicates minimal ligand displacement (B), root-mean-square fluctuation (RMSF) shows stabilized local residues (C), and the radius of gyration (Rg) maintains a compact structure (D). (E) Hydrogen bond analysis reveals stable interaction dynamics. (F, G) The free energy landscape and 2D projection (constructed via principal component analysis) show a concentrated low-energy basin, indicating a predominant, stable binding conformation. (H) Cellular thermal shift assay (CETSA) validates direct target engagement in cells, as Par treatment significantly increases the thermal stability of the UBD protein. Note: Par: Parthenolide; compared to the Control group, ***p < 0.005, ****p < 0.001.

Molecular coupling demonstrated that Par binds stably to a hydrophobic pocket on the surface of the UBD protein, with a favorable binding energy of −6.768 kcal/mol (Figure 5A). Subsequent 100  ns molecular dynamics simulations confirmed the stability of the complex: the RMSD analysis showed minimal ligand displacement (Figure 5B), RMSF indicated stabilized local residue fluctuations (Figure 5C), and the compact radius of gyration (Figure 5D) confirmed structural integrity. The dynamics of hydrogen bonds (Figure 5E) and a concentrated low-energy basin in the free energy landscape (Figure 5F and G) further supported a stable binding mode. The direct interaction was validated in a cellular context using CETSA, where Par treatment significantly improved the thermal stability of the UBD protein (Figure 5H). Together, these results establish Parthenolide as a direct small-molecule inhibitor of UBD.

### Parthenolide induces ferroptosis in ICC cells

**Figure 6.**
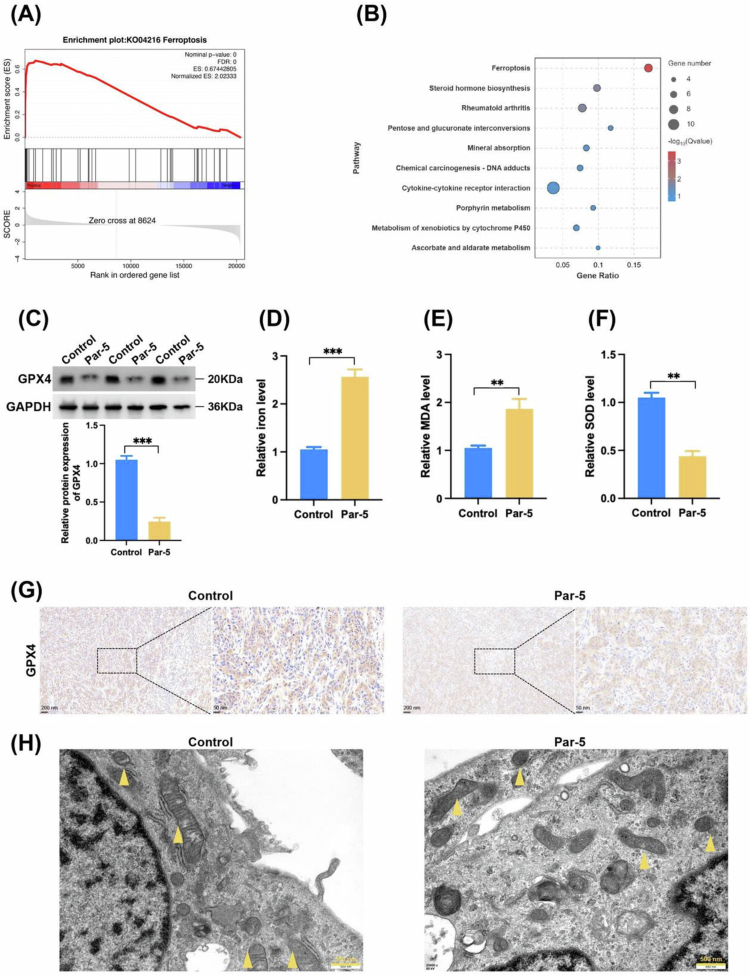
Parthenolide induces ferroptosis in ICC cells. (A) The Gene set enrichment analysis (GSEA) of RNA-sequencing data identifies “ferroptosis” as a significantly enriched pathway in Par-treated HuCCT1 cells. (B) KEGG pathway enrichment analysis further confirms the significant enrichment of the ferroptosis pathway. (C) Western blot analysis shows that Par treatment significantly decreases GPX4 expression (D–F) Increase in intracellular iron levels (D) and lipid peroxidation (measured by malondialdehyde, MDA) (E), accompanied by a decrease in the activity of the antioxidant enzyme superoxide dismutase (SOD) (F). (G) Immunohistochemical staining of subcutaneous xenograft tumors confirms that treatment with Par *in vivo* significantly reduces the expression of the GPX4 protein. (H) Electron microscopy results revealed that Par treatment induced characteristic mitochondrial changes of ferroptosis in cells, including reduced mitochondrial volume, shrinkage, and significantly increased membrane density (Yellow triangle). Note: Par: Parthenolide; compared to the Control group, **p < 0.01, ***p < 0.005.

Further analysis of RNA seq data showed that the “ferroptosis” pathway was significantly enriched by GSEA analysis (Figure 6A). KEGG pathway analysis also showed significant enrichment of the “ferroptosis” pathway (Figure 6B). This suggests that Parthenolide may exert its effect by inducing this novel mode of cell death. Therefore, we further examined the changes in GPX4 after Parthenolide treatment and found that Parthenolide significantly reduced GPX4 expression (Figure 6C). The level of intracellular iron ions was also found to increase significantly after Parthenolide treatment, and the level of the end product malondialdehyde of intracellular lipid peroxidation (MDA) increased significantly, while the activity of the antioxidant enzyme superoxide dismutase (SOD) decreased significantly (Figure 6D–F). Further detection of GPX4 expression in nude mouse tumors also revealed that Parthenolide treatment significantly reduced the expression level of GPX4 (Figure 6G). Electron microscopy results revealed that Par treatment induced characteristic mitochondrial changes of ferroptosis in cells,^24^ manifested as reduced mitochondrial volume, shrinkage, and significantly increased membrane density (Figure 6H). These results indicate that Par can induce ferroptosis in ICC.

### Inhibition of ferroptosis can partially reverse the inhibitory effect of Parthenolide on ICC

**Figure 7.**
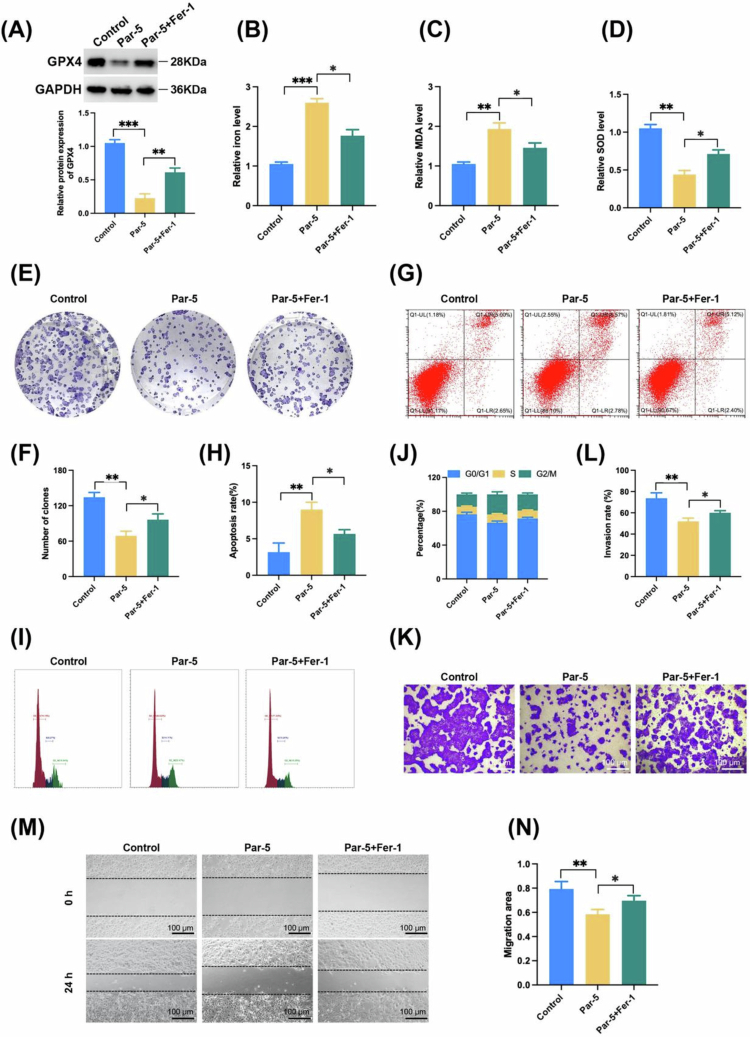
Ferroptosis inhibition can partially reverse the inhibitory effect of Parthenolide on ICC. (A–D) The Ferrostatin-1 ferroptosis inhibitor (Fer-1, 1 µM) co-treatment partially rescues Par-induced ferroptosis in ICC cells, by restoring GPX4 protein expression (A), reducing intracellular iron levels (B) and lipid peroxidation (MDA) (C), and alleviating suppression of SOD activity (D). (E–N) Fer-1 co-treatment partially restores the phenotypic effects of Par. Increase colony formation capacity (E, F), attenuates G2/M phase cell cycle arrest (G, H) and apoptosis (I, J) and mitigates inhibition of cell invasion (K, L) and migration (M, N) induced by Par alone. Note: Par: Parthenolide; *p < 0.05, **p < 0.01, ***p < 0.005.

To clarify whether ferroptosis is a key mechanism for the function of Parthenolide, we conducted salvage experiments using the classic ferroptosis inhibitor Fer-1. The results showed that cotreatment with Fer-1 (1 μM) and Par (5 μM) partially restored the downregulated GPX4 level by Parthenolide (Figure 7A). And it can significantly alleviate iron level upregulation and accumulation of MDA caused by Par alone (Figure 7B and C). This also includes the upregulation of Par and the decrease in SOD levels (Figure 7D). The effect of inhibition of ferroptosis on the inhibitory effect of Par on ICC was then further explored. The results showed that cotreatment with Fer-1 and Par partially restored the ability to form clones inhibited by Par (Figure 7E and F). In addition, Fer-1 partially reversed Par-induced phase arrest of G2/M and cell apoptosis (Figure 7G-J). Similarly, the invasion and migration capacity of cells in the Fer-1 co-treated group was partially restored compared to the Par-treated group (Figure 7K–N). These results indicate that Par-induced ferroptosis is one of the key mechanisms underlying its ability to inhibit ICC cell proliferation, cell cycle arrest, and metastasis. Fer-1 did not completely reverse the effect of Par, suggesting that Par may work synergistically by inducing apoptosis and other pathways.

### UBD is involved in the process of parthenolide promoting ferroptosis.

**Figure 8.**
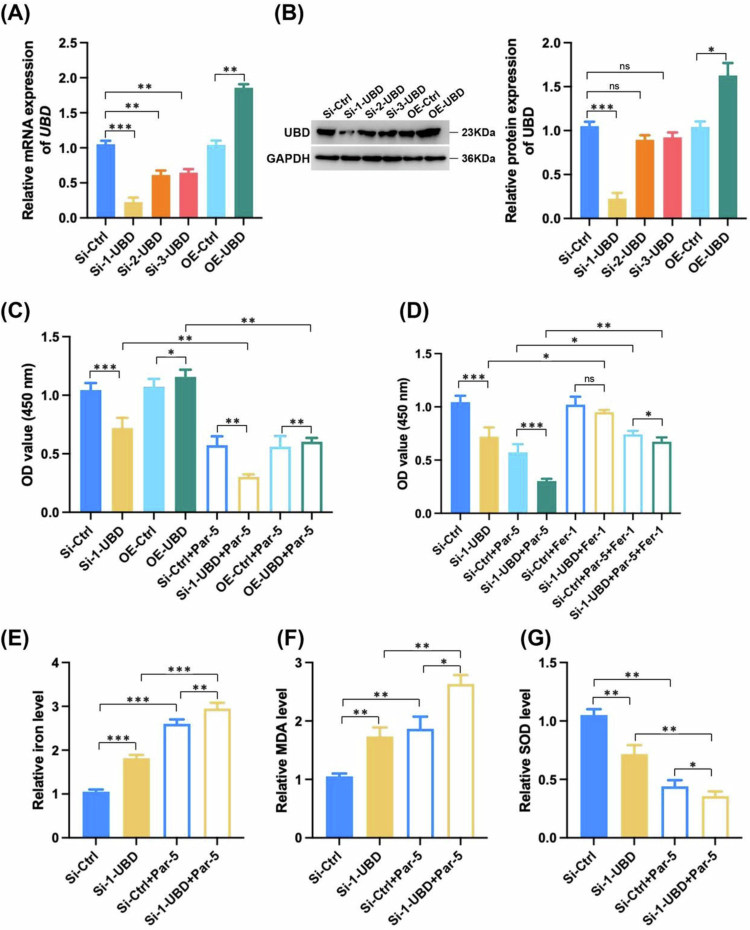
Inhibition of UBD and Parthenolide treatment synergistically promotes ferroptosis in ICC. (A) qRT-PCR analysis of UBD mRNA expression levels in cells transfected with si-UBD constructs and UBD overexpression plasmid. (B) Western blot analysis of UBD protein expression levels in cells transfected with si-UBD constructs and UBD overexpression plasmid. (C) CCK-8 assay results showing cell viability (OD value at 450 nm) in cells with UBD knockdown (Si-1-UBD), overexpression (OE-UBD), and cells treated with the Par. (D) CCK-8 assay results showing cell viability in cells with UBD knockdown, treated with Par alone, or co-treated with Fer-1. (E–G) Quantification of intracellular relative iron level (E), MDA level (F), and SOD activity (G) in Si-Ctrl, Si-1-UBD, Si-Ctrl + Par-5, and Si-1-UBD + Par-5 groups. Note: Par: Parthenolide; *p < 0.05, **p < 0.01, ***p < 0.005, ns: not significant.

To clarify the function of UBD in the process of Parthenolide promoting ferroptosis, siRNA and plasmids were transfected into HUCCT1 cells (Figure 8A and B). Next, we evaluated the effect of UBD on cell viability via the CCK-8 assay. The results demonstrated that UBD knockdown significantly decreased cell viability, whereas UBD overexpression enhanced it (Figure 8C). Par-5 treatment reduced cell viability in the si-Ctrl group, and this inhibitory effect was further exacerbated by UBD knockdown (Figure 8C). Importantly, co-treatment with Fer-1 partially rescued the cell viability loss induced by Par-5 and Si-1-UBD, confirming that UBD could regulate cell survival in a ferroptosis-dependent manner (Figure 8D). UBD knockdown alone significantly increased intracellular iron accumulation and MDA levels, while decreasing SOD activity compared to the si-Ctrl group. Furthermore, when combined with Par-5 treatment, Si-1-UBD transfection led to a more elevation in iron levels and MDA content, as well as a greater reduction in SOD activity, relative to the si-Ctrl + Par-5 group (Figure 8E–G). These findings indicated that UBD was critically involved in Par-5-induced ferroptosis, and the combination of UBD knockdown with Par-5 treatment could exert the most prominent ferroptosis-inducing effect.

## Discussion

Intrahepatic cholangiocarcinoma (ICC) faces serious challenges in clinical treatment due to its high resistance to chemotherapy and invasiveness. This study systematically revealed for the first time that the traditional natural compound, Parthenolide, can effectively inhibit ICC progression in both *in vitro* and *in vivo* models, and innovatively elucidated that Par can activate the Nrf2/HO-1 axis to induce ferroptosis related to inhibition of UBD. This discovery not only provides important evidence for the anti-tumor mechanism of Par but also offers a highly promising new strategy and theoretical basis for the targeted therapy of ICC.

This study confirmed the effective inhibitory effect of Par on ICC proliferation through screening based on an FDA-approved drug library. Furthermore, it was found to inhibit the proliferation, colony formation, and invasion and metastasis capacity of ICC cells. Of particular importance is that Par does not act through a single pathway of death but instead induces classical cell apoptosis and phase arrest of G2/M. This multi-acting mode may help overcome the heterogeneity and drug resistance of tumor cells, providing a basis for the potential of Par as an ICC therapeutic drug. *In vivo* experiments further confirmed its therapeutic value, as Par effectively inhibited tumor growth without showing significant toxicity, demonstrating good therapeutic potential.

Ubiquitin-like protein D (UBD, also known as FAT10) is a protein highly expressed in various cancers.^25^ The function of UBD is to drive tumor cell proliferation and survival by promoting the degradation of key tumor suppressor proteins such as p53.^26^ Studies in colorectal cancer, breast cancer, liver cancer, and other tumors have confirmed that high UBD expression is closely related to tumor progression, drug resistance, and poor prognosis.^26-28^ However, the carcinogenic mechanism, clinical significance, and therapeutic potential of UBD in cholangiocarcinoma are still very limited. This study suggests that Par can inhibition of UBD, thus upregulating NRF2-HO-1 induced ferroptosis. We have supplemented the mechanism of effect of Parthenolide and conducted preliminary research on the expression and role of UBD in ICC.

The decomposition of heme by HO-1 directly produces ferrous ions (Fe2+), leading to an increase in the unstable iron pool in the cell and providing a sufficient iron source for the Fenton reaction, thus catalyzing the production of a large amount of reactive oxygen species of lipids and driving ferroptosis.^29^ The activity of HO-1 requires the consumption of reduced coenzymes within the cell, which may indirectly weaken the overall antioxidant capacity of the cell.^30^ There are multiple HO-1-mediated ferroptosis pathways, among which the Nrf2/HO-1 axis is the most common.^31^ Many natural compounds such as resveratrol and quercetin can induce ferroptosis in tumor cells by activating or regulating this pathway.^10^^,^^32^ Our study also found that Par treatment of cells can upregulate the Nrf2/HO-1 axis and promote ferroptosis. Further consideration could be given to increase the sensitivity of targeted drugs and chemotherapy drugs through the combination of Par and other treatment methods.

Currently, there are no reports on the direct regulatory relationship between UBD and NRF2 in cholangiocarcinoma. In a physiologically stable state, the activity of NRF2 is mainly strictly regulated by proteasomal degradation mediated by ubiquitination, and its protein level is significantly downregulated.^33^ Therefore, promoting NRF2 ubiquitination involves downregulating its protein stability and inhibiting its transcriptional activity. UBD is a ubiquitin-like protein modifier that promotes the recognition and degradation of other proteins by proteasomes.^26^ Therefore, we speculate that after treating cells with Par, the expression of UBD is downregulated, leading to inhibition of the ubiquitination pathway. Subsequently, NRF2 can stably accumulate and enter the nucleus, thus activating the expression of downstream ho-1 genes. The specific mechanism still needs further verification.

This study also has some limitations. The specific post-transcriptional regulation mechanism of Par inhibiting UBD expression still needs to be clarified. If UBD regulates Nrf2 activity, the molecular events involved need further identification. Although animal experiments have shown efficacy and safety, the pharmacokinetic properties of Par remain a challenge for its clinical translation, and future development of its derivatives or novel delivery systems may be necessary. Also, In future studies, more ferroptosis-related indicators and quantitative detection methods will help promote the clinical translation of Par as a ferroptosis inducer.

Despite these challenges, our research has significant implications for translational medicine. It established UBD as a potential drug target for inducing ferroptosis for the first time, while Par can serve as a small-molecule inhibitor of UBD. It is expected to provide a new treatment option for patients with ICC who are resistant to traditional chemotherapy and apoptosis induction therapy.

## Supplementary Material

Supplementary Material Supplementary Table 1.docx

Supplementary Material Supplementary Table 2.docx

Supplementary Material Supplementary Table 3.docx

## Data Availability

Data and materials were included in the manuscript.
