# Supplementary material for: Parthenolide inhibits the progression of intrahepatic cholangiocarcinoma by promoting ferroptosis through inhibiting UBD
Source: Cancer Biol Ther. 2026 Apr 28;27(1):2664327. doi: 10.1080/15384047.2026.2664327 (PMC13134420; doi:10.1080/15384047.2026.2664327)
Supplement: Supplementary Material — Supplementary Table 1.docx [file KCBT_A_2664327_SM6044.docx]

**Supplementary Table 1 the primes for RT-PCR**

| **Gene name** | **Forward Primer** | **Reverse Primer** |
| --- | --- | --- |
| ***HMOX-1*** | **5’-GTGCCACCAAGTTCAAGCAG-3’** | **5’-CACGCATGGCTCAAAAACCA-3’** |
| ***NRF2*** | **5’-CACGGTCCACAGCTCATCAT-3’** | **5’-GGTTGGGGTCTTCTGTGGAG-3’** |
| ***UBD*** | **5’-AGATGGCTCCCAATGCTTCC-3’** | **5’-GCCTCTTTGCCTCATCACCT-3’** |
| ***GAPDH*** | **5’-TTTTGCGTCGCCAGCC-3’** | **5’-ATGGAATTTGCCATGGGTGGA-3’** |
