# Supplementary material for: Parthenolide inhibits the progression of intrahepatic cholangiocarcinoma by promoting ferroptosis through inhibiting UBD
Source: Cancer Biol Ther. 2026 Apr 28;27(1):2664327. doi: 10.1080/15384047.2026.2664327 (PMC13134420; doi:10.1080/15384047.2026.2664327)
Supplement: Supplementary Material — Supplementary Table 2.docx [file KCBT_A_2664327_SM6043.docx]

**Supplementary table 2 the dilution of antibodies**

| **Antibody name** | **Manufacturer and item number** | **Dilution for Western blot** | **Dilution for Immunohistochemistry** |
| --- | --- | --- | --- |
| **HO-1** | **Zenbio, R24541** | **1:1000** | **/** |
| **NRF2** | **Zenbio, R380773** | **1:1000** | **/** |
| **UBD** | **Zenbio, R381482** | **1:1000** | **1:200** |
| **GPX4** | **Zenbio, R381958** | **1:1000** | **1:300** |
| **GAPDH** | **Zenbio, R380646** | **1:2000** | **/** |
