# Supplementary material for: Parthenolide inhibits the progression of intrahepatic cholangiocarcinoma by promoting ferroptosis through inhibiting UBD
Source: Cancer Biol Ther. 2026 Apr 28;27(1):2664327. doi: 10.1080/15384047.2026.2664327 (PMC13134420; doi:10.1080/15384047.2026.2664327)
Supplement: Supplementary Material — Supplementary Table 3.docx [file KCBT_A_2664327_SM6042.docx]

Supplementary Table 3 **The sequences for si-RNA and plasmid**

| **Name** | ****Sequence**** |
| --- | --- |
| **Si-Ctrl** | 5’-UUCUCCGAACGUGUCACGUTT-3’ |
|  | 5’-ACGUGACACGUUCGGAGAATT-3’ |
| **Si-1-UBD** | 5’-GUUCCGAGGAAUGGGAUUUAATT-3’ |
|  | 5’-UUAAAUCCCAUUCCUCGGAACTT-3’ |
| **Si-2-UBD** | 5’-CGAGACUAAGACGGGUAUAAUTT-3’ |
|  | 5’-AUUAUACCCGUCUUAGUCUCGTT-3’ |
| **Si-3-UBD** | 5’-CGGAGAAGCCUCUCAUCUUAUTT-3’ |
|  | 5’-AUAAGAUGAGAGGCUUCUCCGTT-3’ |
| **OE-Ctrl** | 5’-UUCUCCGAACGUGUCACGUTT-3’ |
|  | 5’-ACGUGACACGUUCGGAGAATT-3’ |
| **OE-UBD** | 5’-GUAUGACAACAGCCUCAAGTT-3’ |
|  | 5’-CUUGAGGCUGUUGUCAUACTT-3’ |
